# Manual Therapy as a Management of Cervical Radiculopathy: A Systematic Review

**DOI:** 10.1155/2021/9936981

**Published:** 2021-06-03

**Authors:** Sergio Borrella-Andrés, Isabel Marqués-García, María Orosia Lucha-López, Pablo Fanlo-Mazas, Mar Hernández-Secorún, Albert Pérez-Bellmunt, José Miguel Tricás-Moreno, César Hidalgo-García

**Affiliations:** ^1^Health Sciences Faculty, University of Zaragoza, Zaragoza 50009, Spain; ^2^Physiotherapy Research Unit, University of Zaragoza, Zaragoza 50009, Spain; ^3^Department of Basic Sciences, Faculty of Medicine and Health Sciences, Universitat Internacional de Catalunya, Sant Cugat del Vallés 08195, Spain; ^4^ACTIUM Functional Anatomy Group, Universitat Internacional de Catalunya, Sant Cugat del Vallés 08195, Spain

## Abstract

**Background:**

Cervical radiculopathy is defined as a disorder involving dysfunction of the cervical nerve roots characterised by pain radiating and/or loss of motor and sensory function towards the root affected. There is no consensus on a good definition of the term. In addition, the evidence regarding the effectiveness of manual therapy in radiculopathy is contradictory.

**Objective:**

To assess the effectiveness of manual therapy in improving pain, functional capacity, and range of motion in treating cervical radiculopathy with and without confirmation of altered nerve conduction.

**Methods:**

Systematic review of randomised clinical trials on cervical radiculopathy and manual therapy, in PubMed, Web of Science, Scopus, PEDro, and Cochrane Library Plus databases. The PRISMA checklist was followed. Methodological quality was evaluated using the PEDro scale and RoB 2.0. tool.

**Results:**

17 clinical trials published in the past 10 years were selected. Manual therapy was effective in the treatment of symptoms related to cervical radiculopathy in all studies, regardless of the type of technique and dose applied.

**Conclusions:**

This systematic review did not establish which manual therapy techniques are the most effective for cervical radiculopathy with electrophysiological confirmation of altered nerve conduction. Without this confirmation, the application of manual therapy, regardless of the protocol applied and the manual therapy technique selected, appears to be effective in reducing chronic cervical pain and decreasing the index of cervical disability in cervical radiculopathy in the short term. However, it would be necessary to agree on a definition and diagnostic criteria of radiculopathy, as well as the definition and standardisation of manual techniques, to analyse the effectiveness of manual therapy in cervical radiculopathy in depth.

## 1. Introduction

Cervical radiculopathy (CR) can be defined as a disorder involving dysfunction of the cervical nerve roots, commonly presenting with pain radiating from the neck towards the root affected [1]. Commonly, there is no agreement on the definition, given that it has also been defined as neck and shoulder pain combined with loss of sensory and motor function [2, 3]. Nevertheless, Thoomes et al. [4] proposed a new definition of CR as a radiating pain in the arm with motor, reflex, and/or sensory changes (such as paraesthesia or numbness), provoked by neck posture(s) and/or movement(s).

The incidence of CR has been established to be between 63.5 and 107.3 per 100.000 people per year [5], with the C6 and C7 segments being the most affected [6]. As for the treatment given, there are 2 main approaches: the conservative option and the surgery. Clinical guidelines from 2011 and 2018 recommend exercise, manual therapy, and nonsteroid anti-inflammatory drugs (NSAIDs) as the first line of treatment [7, 8]. If this first treatment option gives no relief within 4 to 8 weeks [9], analgesic/anti-inflammatory drugs would be injected, recurring to surgery if necessary. Taking this route normally depends on how severe the patient's symptoms are. Evidence has also been found that both the surgical and the conservative approaches produce improvements by 2 years, with no statistically significant differences between them [10]. Other authors concluded that evidence on manual therapy was inconclusive or low levelled, and one single intervention cannot be recommended [11–13].

Previous systematic reviews have provided indications that exercise is effective in treating CR, whether associated or not to manual therapy [14, 15], manual or mechanical traction [16], and high-velocity/low-amplitude manipulations [17]. All the reviews coincide in the need to establish a treatment protocol and carry out studies having larger samples. However, the Task Force on Neck Pain and Its Associated Disorders (TFNPAD) concluded that there was insufficient evidence for recommending appropriate treatment for CR [18]. Consequently, the evidence as to the effectiveness of manual therapy in radiculopathy, regardless of the region, is contradictory.

Besides, without uniform CR diagnosis criteria, one might compare treatments with samples possibly having motor and/or sensory disorders to others in which the individuals have pain without altered nerve conduction velocity, with prognoses that are probably different. Despite conflicting evidence at the use of nerve conduction as a diagnostic criterion [7, 19], it has shown moderate diagnostic accuracy [20] and moderate-excellent specificity for radiculopathy [20, 21]. Guidelines from the American Association of Neuromuscular and Electrodiagnostic Medicine (AANEM, previously American Association of Electrodiagnostic Medicine when published) recommend that for an optimal evaluation of a patient with suspected radiculopathy, a needle EMG screen of a sufficient number of muscles and at least one motor and one sensory nerve conduction study (NCS) should be performed in the involved limb [22]. NCSs are necessary to exclude polyneuropathy [22]. To date, no systematic review has been found that includes the idea of manual therapy effectiveness in CR, in relation to altered nerve conduction measured with nerve conduction tests.

The objective of our systematic review is thus to verify the effectiveness of manual therapy in CR cases with and without confirmation of altered nerve conduction—using electromyography or electroneurography—in pain, disability, function, and range of movement, in comparison to other physiotherapy techniques used in the approach to CR.

## 2. Materials and Methods

### 2.1. Protocol and Recording

Our review was carried out in agreement with the PRISMA statement checklist [23] and the criteria of the Cochrane Handbook for Systematic Reviews of Interventions [24]. This systematic review was registered on the Open Science Framework digital platform: https://osf.io/zgdym/.

### 2.2. Information and Search Sources

**Figure 1:**
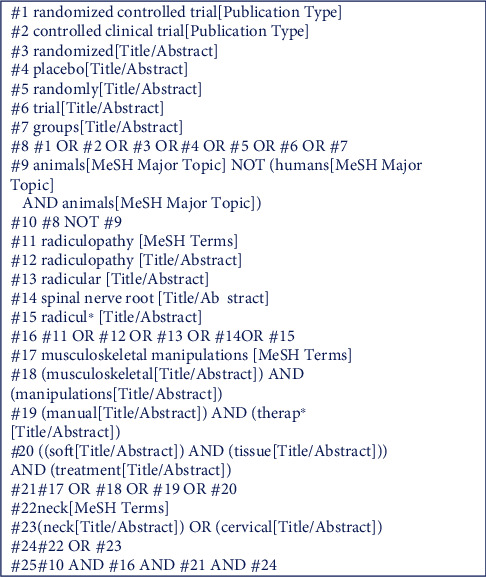
Search strategy.

Search strategy was developed following advice of an information specialist (MHS) from the research group, with two-year experience in conducting systematic reviews. The following reference sources were searched: PubMed, Web of Science, Scopus, PEDro, and Cochrane Library Plus. The search terms were divided into 4 categories, as recommended by the Cochrane Back and Neck Group [25]. The first category was established to search for the types of studies to include: randomised controlled trial or controlled clinical trial. The second and third categories searched specifically for the condition (radiculopathy) and for manual therapy actions (musculoskeletal manipulations), while the fourth category served to limit the search to the cervical area (neck). These 4 searches were combined to obtain the results.  Figure 1 presents a detailed search strategy, featuring the database search in PubMed, from which the rest of the databases were searched, adapting the searches to the requirements of each database.

### 2.3. Eligibility Criteria

The inclusion criteria were as follows: (1) randomised controlled clinical trials on humans; (2) patients with CR, identified and diagnosed by clinical criteria (arm, neck, scapular or periscapular pain and paresthesias, numbness and sensory changes, weakness, or abnormal deep tendon reflexes in the arm) [7] or by using references tests (shoulder abduction, Spurling's test) [7], electromyography or electroneurography, and the clinical criteria for radiculopathy diagnosis which had to be explained completely; (3) assessment of the effectiveness of manual therapy in CR, with no distinction between manipulations, mobilisations, and soft tissue treatment or combined with other techniques; (4) pain, disability, or Short Form-36 Health Survey (recommended by the North American Spinal Association in their CR guidelines [7]; (5) studies published in English, French, and Spanish; and (6) studies published in the past 10 years.

We excluded articles that (1) used another approach, except if they used a comparison to the manual therapy technique or were accompanied by it, and (2) with patients with neck and arm symptoms without a CR diagnosis.

### 2.4. Study Selection

Two researchers (IMG and SBA) selected the studies independently. If they disagreed on the study selection, the final decision was made by a third researcher (CHG).

### 2.5. Data Mining Process

Data on sample size, criteria for radiculopathy diagnosis, type of intervention, treatment protocol, follow-up, variables studied, and main results were included. With regard to the variables, the primary results, secondary results, and adverse effects were recorded.

### 2.6. Risk of Bias in the Individual Studies

Two independent researchers assessed methodological quality of the studies using the PEDro scale; there was a third researcher to settle cases of doubt or disagreement. The PEDro scale [26] evaluated 11 items, scoring each item as 1 or 0, depending on whether the item fulfilled study criteria or not, respectively. External validity was assessed using Item 1, internal validity using Items 2 through 9, and interpretability of the results using Items 10 and 11. The first item was not taken into account for the final score, which could be a maximum of 10 points. Each article was classified according to the score obtained: “high quality” if its score was ≥6, “moderate quality” with a score of 4-5, and “low quality” if the score was <4.

Also, the RoB2 tool was performed. It is the second version of the Cochrane tool to assess the risk of bias in clinical trials. The biases are evaluated in 5 domains: (1) randomization process, (2) effect of being assigned to intervention, (3) missing outcome data, (4) measurement of the outcome, and (5) reported results. Within each domain, 1 or more questions must be answered. These answers lead to the judgements of “low risk of bias,” “some concerns,” or “high risk of bias” [27].

## 3. Results and Discussion

### 3.1. Study Selection

**Figure 2:**
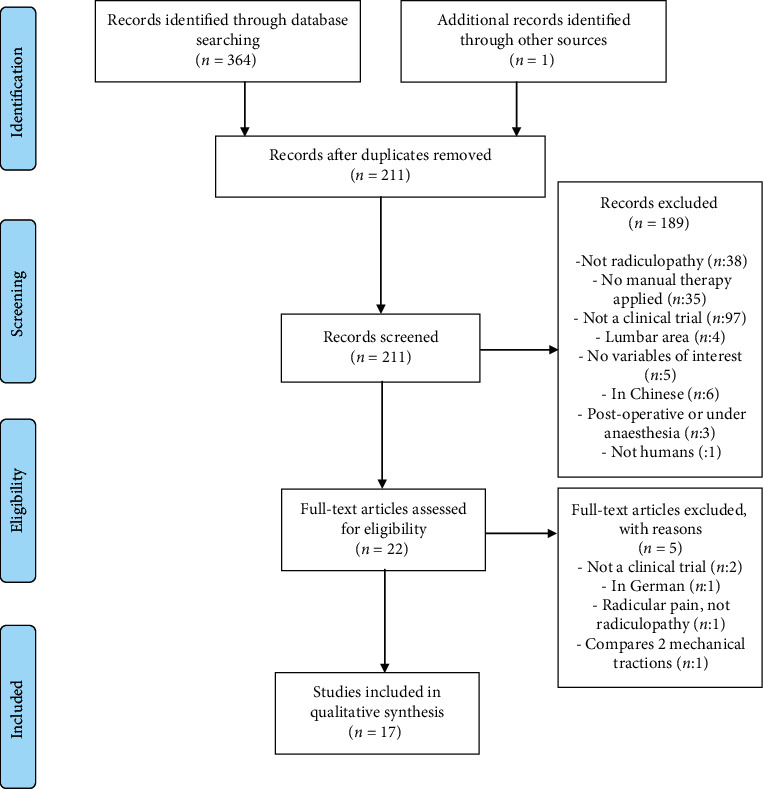
PRISMA flow diagram.

The initial literature search yielded a total of 365 studies. After eliminating the duplicates, there were 211 articles left. Filtering on title and abstract yielded 22 articles for complete text reading, of which 5 were eliminated, because they did not meet any of the inclusion criteria. Seventeen studies were selected for final inclusion. The PRISMA flow diagram (Figure 2) illustrates the process.

### 3.2. Methodological Quality Assessment

The methodological quality of the trials included in the revision is summarised in Table 1, indicating if each of the PEDro Scale items is fulfilled or not. Of the 17 studies selected, 9 had an overall score of high quality [28–36]. Three studies had a moderate overall score [37–39] and 5 studies a low score [40–44].

Three studies [38, 40, 41] did not assign patients randomly, and in the majority of the studies, patients were assigned with an inadequate concealment method. In four [40, 42–44] of the 15 studies, the intervention groups were not compared at the beginning of the study. All studies reported the lack of therapist blinding and the lack of patient blinding. Only 3 studies [33, 35, 36] reported blinding of the assessors to the group assignment of the patients.

**Figure 3:**
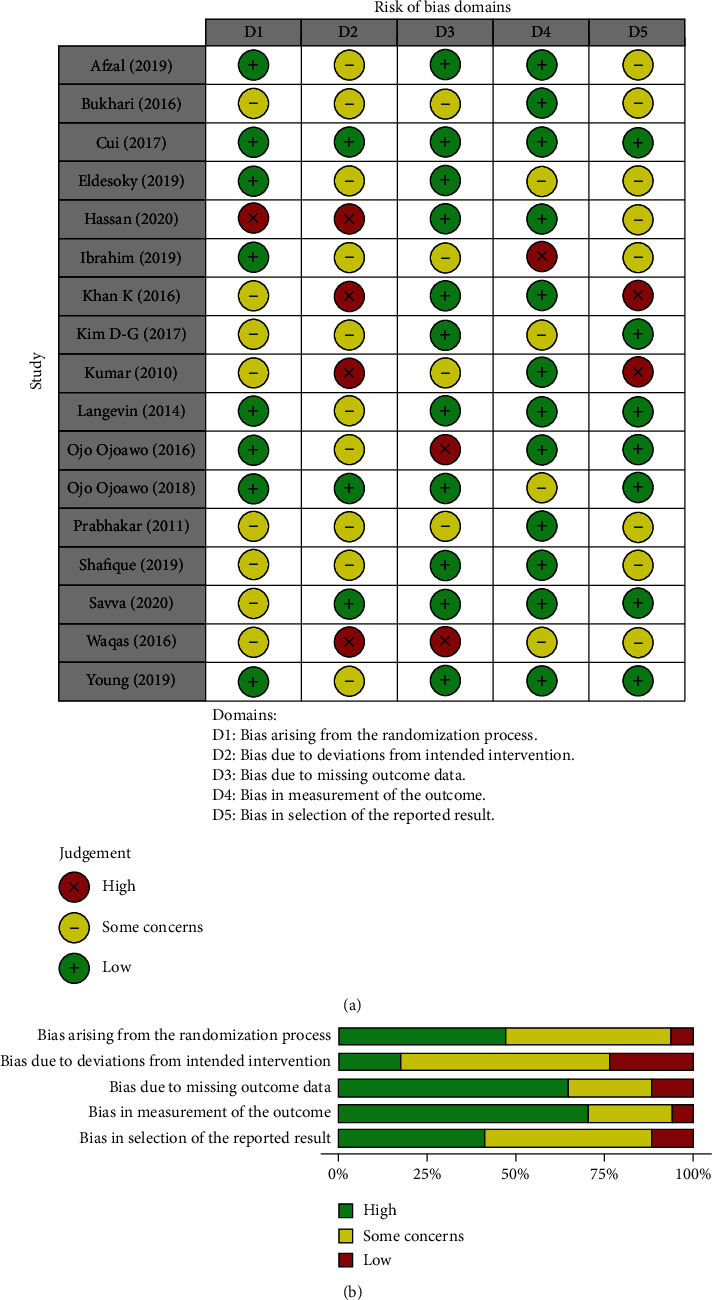
(a) Summary of risk of bias 2.0. and (b) Risk of bias 2.0. graphs.

The RoB2 tools showed that the aspects with the worst methodological quality in all the studies are related to deviations from the intended intervention. Reported data and variable measurements seem to have the best methodological quality in all the studies, with 75% of the studies meeting these criteria (Figures 3(a) and 3(b)), even though the majority of studies had good/moderate methodological quality.

### 3.3. Clinical Trial Characteristics

Characteristics of the studies are presented in Table 2. There are a total of 1.183 patients. In all the studies, patients having symptoms compatible with a CR diagnosis who reported radiation of symptoms to an upper limb were discussed. CR diagnosis was established in all studies using the clinical findings, mentioning pain radiation or neural symptoms in the upper limb. None of the studies evaluated diagnosed radiculopathy using electromyography or electroneurography, and magnetic resonance was only used in 2 of the studies [30, 31] to reveal the possible cause of neural compression.

Nerve conduction velocity was analysed in just one study, using evoked potentials [31]. This test was performed as a measurement variable, not as a diagnostic criterion. In 8 of the studies assessed, the prediction rule formulated by Wainner et al. [45] was applied; this rule refers to a specificity of 94%-100% in diagnosing CR [30, 33, 35, 36, 38, 39, 43, 44].

The tools used to measure the study outcomes were similar in all the studies. The intensity of perceived pain was measured using a Visual Analogue Scale (VAS) in 8 studies [30, 33, 35, 36, 38, 39, 43, 44], while the Numeric Pain Rating Scale (NPRS) was used in the rest [30, 32, 34–36, 38, 39, 42, 43]. In 11 of the studies [28–32, 34–39, 42, 43], cervical disability was measured with the Neck Disability Index (NDI). As a secondary indicator of technique effectiveness, cervical mobility was measured in 6 of the studies [34, 35, 38–40, 43]. Likewise, quality of life was measured in just 1 of the studies [37], using the SF-36 Health Survey.

All the studies analysed reported a statistically significant improvement in the variables of pain and cervical disability index in the manual therapy intervention group, regardless of the protocol and the manual therapy technique applied. The manual therapy techniques of choice were divided among cervical manipulations, thoracic manipulations, cervical mobilisation towards the intervertebral foramen opening, cervical traction, and neural mobilisations. The techniques with the best results were those whose objective was to increase the intervertebral foramen area by transverse mobilisations in the indicated segments. These techniques had been applied with positive results in 4 of the studies [28, 29, 41, 43]. However, one of them did not find differences with the control group [36]. Both the cervical and thoracic manipulation techniques yielded satisfactory results. Specifically, two studies on thoracic manipulation [32, 34] and one on cervical manipulation combined with other manual techniques [37] found the results to be satisfactory.

In contrast, manual traction and neural mobilisation were the least satisfactory techniques. Traction obtained worse results with respect to the intervertebral foramen “openings” and mobilisations [28, 40], but it was superior to conventional treatment [35, 44]. Neural mobilisation techniques also obtained worse results compared against the intervertebral foramen “opening,” mobilisation, manipulation, and traction [40, 43] and similar results to conventional treatment [33]; better results were found if it was combined with traction [35, 38]. Cervical mobilisation techniques according to MacKenzie [40], to Maitland [32, 39], and to Kaltenborn [39] were applied in 3 of the studies.

The duration of the intervention and the guidelines for establishing the duration were reported in all the articles, except for the study of Waqas et al. [32]. Times and repetition of application for each technique varied among studies, from 3 repetitions in a series [28, 29] up to 30 repetitions per 3 series [43] for the foramen openings. In the case of traction, times of application ranged from 10 [38, 42] to 20 minutes [37, 40]. The study of Bukhari et al. [42] was the only one to establish 10 repetitions for manual traction. As for neural mobilisations, these were applied from nerve gliding exercises with 10 repetitions [33], to nerve gliding repeated for 10 minutes [38].

The number of sessions applied was established as 8 and 12 in most of the articles. Young et al.'s study [34] was the study in which the fewest sessions were given (a single manipulation session), while in that of Kim et al. [38], the most were applied (24 sessions in 8 weeks). In the majority of the studies, a mean of 2-3 sessions a week was applied. The exceptions were the study of Kumar [40], in which 10 sessions on consecutive days were given, and that of Khan et al. [44], in which 6 sessions a week were applied.

The results of this review should be taken as short-term results, given that the follow-ups were generally performed half-way through the study and/or when the intervention period ended. Just a single article [37] had a medium-term follow-up, taking measurements up to almost 6 months after the intervention.

None of the studies revealed mild or serious adverse effects in the application of the techniques chosen.

## 4. Discussion

The objective of this review has been to ascertain the effectiveness of manual therapy in handling CR cases with and without confirmation of altered nerve conduction. In previous studies, the effectiveness of exercise in CR [14] and the interest in combining manual therapy with exercise has been demonstrated. The combination improved function, range of movement, and pain. However, it has been impossible to establish which manual therapy techniques are the most effective [15].

There is a lack of scientific research on applying manual therapy techniques (such as manipulations and mobilisations) in this pathology [16], as well as a lack of rigour in describing the manual therapy techniques used. A previous review showed a lack of evidence for the effectiveness of manual therapy in patients with CR [13], due to low-quality evidence or interventions studied only once. A noninvasive therapy in cervicobrachial pain has been previously revised with inconclusive evidence for the effectiveness of noninvasive management [11]. Considering previous reviews, several new studies have appeared and been included in this review, contributing to the new evidence regarding CR and manual therapy.

Degenerative signs in imaging tests need to be correlated with the clinical findings of the pathology for accurate CR diagnosis [46]. Therefore, our study has considered the relationship between patients with symptomatology compatible with CR and a medical diagnosis with imaging tests or nerve conduction tests.

All the studies analysed reported a statistically significant improvement in the variables pain and cervical disability index. Such improvements did not depend on the manual therapy protocol and the technique applied.

This review reveals the need to establish reference criteria for diagnosing “CR” cases, attempting to avoid a catch-all approach. None of the studies included performed electromyography or electroneurography to determine nerve conduction alteration, and only 2 of the studies carried out magnetic resonance imaging to demonstrate possible structural nerve root compromise [30, 31]. It could be explained because of the conflicting evidence of using a conduction test as a diagnostic criteria [7, 19].

The studies include patients with symptomology similar to radicular conditions but not objectively cases of radiculopathy, due to the used diagnostic criteria. Myofascial trigger points, faceted radiated pain, and distal neural compressions might simulate similar symptomology.

Wainner et al. [45] formulate a prediction rule for diagnosing CR cases, achieving specificity of 94%-100% depending on the positivity of four diagnostic tests. However, none of these four tests involves loss of neural conduction in the form of loss of sensitivity and/or motor function and this prediction rule was based on a limited sample of 16 subjects with mild and moderate C6 and C7 CR and 57 controls. Schmid et al. [47] recommended adding pain and thermal sensibility to tactile and motor testing to include A*δ* and C nerve fibers within the evaluation. A new standard definition of radiculopathy is needed, along with relevant diagnostic criteria.

Differentiation should be established between radiculalgia and radicular pain without altered nerve conduction velocity and radiculopathy involving loss of nerve conduction velocity (symptoms and/or signs of neural-origin sensory or motor loss). Similarly, Shacklock [48] defined CR neural management based on the intensity of the symptoms and the presence of neurological symptoms. This classification also guides professionals in the tests that have to be performed to alleviate or provoke patient symptoms more or less easily, as well as in the treatment techniques to use. For example, in patients classified as “Level 1,” neural mobilisation techniques in tension might be contraindicated, because they may generate symptoms and could be counterproductive [48].

A combination of diagnostic criteria based on clinical findings compatible with radiculopathy, the prediction rule of Wainner et al. [45], clinical tests for various sensory and/or motor involvement, imaging tests such as magnetic resonance, and clinical tests for nerve conduction alteration should be performed. Thoomes et al. [19] propose a “combination of Spurling's, axial traction, and an Arm Squeeze test to increase the likelihood of a cervical radiculopathy, whereas a combined results of four negative neurodynamics tests and an Arm Squeeze test could be used to rule out the disorder.” Nowadays, the usefulness of electrodiagnosis is still under debate; the NASS referred insufficient evidence to make a recommendation for or against EMG for patients with unclear diagnosis after clinical examination and MRI [7].

Given that there is symptomology presumably caused by radiculopathy, the techniques that offered the best results were those searching for the intervertebral foramen opening by using pressure or transverse oscillations. The neurodynamic techniques yielded the worst results; the parameters applied were a bit unclear, categorising the neurodynamic technique used as neural gliding exercises [38], mobilising only the elbow and wrist, and probably putting tension on the root and not performing a gliding exercise [48, 49]. Rade et al. [50] showed the conus medullaris displacement in all three planes when unilateral and bilateral SLR were applied. This represents a protective mechanism which preserves the spinal cord and lumbar neural roots from excessive strain. This could allow gliding treatment of the affected nerve root with the help of the mobilisation of the contralateral neurodynamic test. It would be interesting to observe the potential gliding effect of contralateral neurodynamic test on the nerve root on CR patients. A recent study has shown the effect of contralateral cervical lateral glide on CR patients [51], in which longitudinal median nerve excursion differed significantly between patients with CR and asymptomatic volunteers at baseline. This difference was no longer present after 3 months of conservative physiotherapy management.

This fact has led us to analyse the importance of establishing exact parameters for the technique applied and the technical development of its application. Because manual techniques are involved, the variables to control may depend on performance. Reflecting the performance parameters appropriately in the studies is recommended so that the results can be reproduced and extrapolated to the clinical setting. This is something at which the majority of studies has failed, naming the technique applied but not explaining it in detail. Our recommendation would be to avoid describing manual techniques in general, based only on the name of the used methodologies (Maitland, Kaltenborn) but without identifying precisely the technique. Our recommendation is to describe the procedures precisely using the TIDieR checklist to make sure they can be reproduced [52].

Treatment times and doses varied from some studies to others, within similar techniques. The mean was 2-3 sessions a week, applying approximately 8-12 sessions. There are no previous studies dealing with the number of sessions needed for conservative treatment of CR cases. These first guidelines in treatment times are considered interesting; in future studies, the mean treatment times with other approaches can be analysed to compare the time indication of each of the techniques.

As for the number of sessions proposed, thoracic manipulation might be one of the first techniques to apply because it has demonstrated beneficial results from a single session [34] and can be complemented with intervertebral foramen openings in following sessions. These 2 techniques showed the best results, obtained in 2 of the studies with a sufficient sample size [28, 41]. Cui et al. [37] showed high effectiveness in their study involving a multimodal approach to CR based on movement and acupuncture. These results can be considered for clinical application, seeking an approach to all the dysfunctional components found in the assessment by combining different manual therapy procedures.

The results presented in this review should be considered at short term, as the studies involve only short-term follow-up. Manual therapy can be proposed as a way to provide relief when the symptoms might be related to radiculopathy, but its long-term effectiveness has been impossible to establish.

The 3 indicators recommended by clinical guidelines for CR [7] have been analysed as primary variables. It would also be of interest for future studies to include the new functional impact scale, developed specifically for CR [53], as a measurement variable. After seeing the results of this systematic review, it is obvious that there is a need to make the definition of manual therapy techniques and the inclusion criteria more homogeneous and to provide greater detail in defining technique application parameters. This would make possible to advance in future meta-analyses and reviews, as it has been achieved in the approach to CR cases using exercise [14], by establishing more favourable parameters, techniques, and doses.

Our systematic review is strongly limited by the variability of the inclusion criteria of patients with CR and of the manual therapy techniques used, given that these have been very different or poorly explained in the majority of the studies. This means that it has been impossible to analyse the results specifically in the application of each of the techniques. Another limitation is that there may be references available besides the ones in Spanish, English, and French that have not been considered due to the language requirements established in our inclusion criteria. Finally, in the absence of a Cochrane Information Specialist, an information specialist of the group with a two-year experience was available that might not be able to fulfil all the functions.

## 5. Conclusions

We cannot state that manual therapy is effective in treating CR cases confirmed by EMG or ENG tests, due to a deficit in the diagnostic criteria used in the scientific literature. Without an EMG and ENG confirmation, a multimodal approach that includes manual therapy seems to be the most effective short-term approach. The methodological deficiencies and the lack of follow-ups in the studies have to be treated cautiously. It is therefore necessary to establish greater scientific evidence with high quality with larger sample sizes and longer follow-up times, in which initial treatment parameters are established for each of the manual therapy techniques. There is currently a lack of standardisation of diagnostic processes and of treatment in this pathology, even though manual therapy has been shown to be effective in dealing with CR cases having a diagnosis based on clinical criteria.

## Figures and Tables

**Table 1:** Methodological quality according to the PEDro scale.

Study	1	2	3	4	5	6	7	8	9	10	11	Total	Quality
Afzal (2019)	X	X	X	X				X		X	X	6	High
Bukhari (2016)	X	X						X			X	3	Low
Cui (2017)	X	X		X				X		X	X	5	Moderate
Eldesoky (2019)	X	X	X	X				X	X	X	X	7	High
Hassan (2020)	X	X		X			X	X		X		5	Moderate
Ibrahim (2019)	X	X	X	X			X	X	X	X	X	8	High
Khan K (2016)	X	X									X	2	Low
Kim D-G (2017)	X			X				X	X	X	X	5	Moderate
Kumar (2010)	X									X	X	2	Low
Langevin (2014)	X	X	X	X			X	X	X	X	X	8	High
Ojo Ojoawo (2016)	X	X	X	X				X	X	X	X	7	High
Ojo Ojoawo (2018)	X	X	X	X				X	X	X	X	7	High
Prabhakar (2011)	X			X						X	X	3	Low
Shafique (2019)	X	X								X	X	3	Low
Savva (2020)	X	X	X	X			X	X	X	X	X	8	High
Waqas (2016)	X	X		X				X	X	X	X	6	High
Young (2019)	X	X	X	X				X	X	X	X	7	High
Mean												5.5	

**Table 2:** Study characteristics.

Study	Subjects, no. (M/F) [Age range]	Selection criteria	Intervention	Protocol	Outcomes	Main results
Afzal (2019)	40 (17/23)	Unilateral pain in upper limb, paraesthesia w/o numbness. RMN 3 of 4 items +: Spurling, facet distraction, ULTT1, ipsilateral rotation < 60° EMG/ENG: no	G1: openings G2: manual cervical traction G3: G1 + G2	Treatment: 3 ssn/3 weeks G1: 10 reps/3 series G2:10 sec traction/5 sec rest (10 min) All: 15 min heat before Follow-up: ending	NDI NPRS Cervical ROM Patient-specific PSFS	Statistically significant improvement in the 3 groups in all the outcomes

Bukhari (2016)	42 (-) [20-70]	“Evident symptoms of cervical radiculopathy” EMG/ENG: no	G1: mechanical traction G2: manual traction Both: segment mobilisation & exercises	Treatment: 3 ssn/6 weeks G1: 10 sec traction/5 sec rest (10 min) G2: 10 sec traction/5 sec rest (10 reps) Anteroposterior mobilisation: 5 sec/10 reps Follow-up: ending	NPRS NDI	Significant changes in both groups G1 > G2, no comparison between groups

Cui (2017)	359 (25%/75%) [18-65]	Neurologist diagnosis. Pain in 1 or more dermatomes, weakness, & hyporeflexia EMG/ENG: No	G1: mobilisation plus Shi-style cervical manipulation G2: mechanical cervical traction	Treatment: 6 ssn in 2 weeks Mobilisation plus Shi-style cervical manipulation: neck, back, thoracic massage, & UL movement (3‐6 times) + cervical manipulation + pressure point auriculotherapy (30 sec/point) Mechanical cervical traction: 20 min Follow-up: ending, weeks 2, 10, & 22 postfinal Tx	NDI VAS SF-36	Statistically significant improvement in both groups, G1 > G2 in VAS & NDI NDI difference disappears at 22 weeks VAS: difference disappears at 10 weeks

Eldesoky (2019)	50 (-) [18-60]	Unilateral hernia diagnosis in C5 or C6 confirmed using RMN Pain, paraesthesia, or numbness in the C6 or C7 dermatome. Cervical or periscapular pain, cervical rigidity, alteration in dermatome sensitivity, weakness, & muscular atrophy in myotome. Lack of tendon reflexes for more than 3 months	G1: conventional Tx + cervical mobilisation (MC) G2: conventional Tx	Treatment: 3ss/4 weeks Conventional Tx: ultrasound (5 min) + stretching & strengthening MC: posteroanterior & oscillatory rotations in C6 & C7 (10 reps/30 sec/technique) Follow-up: upon ending Tx & at 4 weeks post-Tx	VAS NDI SEP	Both groups: statistically significant improvement in all the outcomes; G1 statistically superior

Hassan (2020)	40 (27/13) [30-70]	Neck pain less than 8 on NPRS & numbness/paraesthesia and/or pain in the arm or the hand. Positive findings of cervical radiculopathy on X-rays 3 of 4 items +: Spurling, facet distraction, ULTT1, ipsilateral rotation < 60° EMG/ENG: no	G1: heat + TENS + oscillatory mobilisations (Maitland) G2: heat + TENS + mobilisation of static stretching (Kaltenborn)	Treatment: 7 ssn/2 weeks G1: heat + TENS (10 min) + Maitland mobilisations (3 × 15 reps) G2: heat + TENS (10 min) + Kaltenborn mobilisation (3 series) Follow-up: ending	NPRS NDI Cervical ROM	Significant improvements in all variables in both groups G1 > G2: significant differences for all the outcomes except for NPRS & inclinations

Ibrahim (2019)	40 (-) [20–40]	Radiated pain in 1 of the arms 3 of 4 items +: Spurling, facet distraction, ULTT1, ipsilateral rotation < 60° EMG/ENG: no	G1: conventional Tx G2: conventional Tx + neurodynamic Tx	Treatment: 3 ssn/3 weeks Conventional Tx: manual traction (15 sec/10 reps/3 series) + stretching (15 sec/10 reps/3 series) + infrared (20 min) Neurodynamic Tx: ULTT gliding (10 reps) + ULTT tensioning (10 sec) x 2 Follow-up: ending	VAS Pressure strength	Statistically significant changes in both groups, without significant differences between them

Khan (2016)	100 (50/50) [25-55]	Cervical radiculopathy neurologist's diagnosis 3 of 4 items plus: Spurling, facet distraction, ULTT1, ipsilateral rotation < 60° EMG/ENG: no	G1: conventional Tx G2: conventional Tx + cervical traction	Treatment: 6 ss/week. 12ss Conventional Tx: exercises (25 reps/twice a day) Continuous TENS (20 min) Heat Cervical traction: 10 sec/5 sec rest (20 min) Follow-up: ending	VAS	G2 > improvement in VAS

Kim (2017)	30 [25-60]	Pain radiation in 1 of the ULs 3 of 4 items +: Spurling, facet distraction, ULTT1, ipsilateral rotation < 60° EMG/ENG: no	G1: sustained manual cervical traction + neural mobilisation G2: manual cervical traction	Treatment: 3 ss/8 weeks Both: heat (20 min) & TENS (15 min) Cervical traction: 1 min/30 sec rest (10 min) Neural mobilisation: median gliding exercises (combines flexion-extension in wrist & elbow) (10 min) Follow-up: weeks 4 & 8 post-Tx	NPRS NDI ROM FME	Statistically significant improvement in the 2 groups. G1 > G2 statistically significant in all the outcomes

Kumar (2010)	30 (10/20) [25-68]	Diagnosis by neurologist or traumatologist Spontaneous pain in the neck, with radiation in the arm, forearm, hand EMG/ENG: no	G1: McKenzie mobilisation G2: neural mobilisation G3: diathermia & mechanical cervical traction	Treatment: 10 sessions in 3 weeks G1: 10-15 openings (1 rep/5 sec) G2: 100 Hz/50 *μ*s/30 min G3: 6-8 sec/rep, 5 reps (flexion, extension, inclination, rotation) Heat: 20-25 min cervical posterior Follow-up: weeks 3 & 6 post-Tx	VAS Cervical ROM: scalar method	VAS: all with statistically significant reduction (G1 > G3 > G2) ROM: improvement in the 3 Adverse effects: not reported

Langevin (2014)	36 [18-65]	Pain in superior or periscapular limb, paraesthesia or numbness > 3 meses 3 of 4 items +: Spurling, facet distraction, ULTT1, ipsilateral rotation < 60° EMG/ENG: no	G1: TM & exercise to open intervertebral foramen G2: TM & exercise	Treatment: 8 ss in 4 weeks G1: MT 10 reps/30 sec→foramen opening + manual traction (5 min)+ at-home exercise G2: equal to G1 without foramen opening objective Follow-up: weeks 4 & 8	NDI NPRS QuickDASH	Statistically significant difference in both groups in the 3 outcomes. No differences between groups

Ojo Ojoawo (2016)	25 (15/12) [-]	Traumatologist diagnosis EMG/ENG: no	G1: TOP + conventional Tx G2: conventional Tx	Treatment: 3ss/4 weeks. Conventional Tx: massage (3 min) + cryotherapy (7 min) + isometric exercises TOP: 20 sec/3 reps Follow-up: weeks 2 & 4	VAS NDI	Significant improvement in both groups. G1 > G2 statistically significant in VAS

Ojo Ojoawo (2018)	75 (40/35) [-]	Cervical pain radiated in the arm. Positive test in: Pinched nerve, posteroanterior pressure, Spurling test & positive Valsalva EMG/ENG: No	G1: cervical traction (CT) (door anchor) + exercise + cryotherapy G2: transversal oscillatory pressure (TOP) exercise + cryotherapy G3: exercise + cryotherapy	Treatment: 2 ss/week/6 weeks G1: TC 15 min G2: TOP 20 sec/3 reps G3: isometrics + stretching 3 ss/week/6 weeks Follow-up: weeks 3 & 6 Tx	VAS NDI	Statistically significant improvements in all groups VAS: G2 change quicker (weeks) & greater at 6 weeks NDI: no statistically significant differences; yes, greater percentage of change in G1 & G3

Prabhakar (2011)	75 (48%/52%) [20–50]	Diagnosis of cervical spondylosis with subacute radiated pain in upper limb EMG/ENG: no	G1: heat + dynamic cervical opening + isometrics G2: heat + TENS + isometrics G3: heat + isometrics	Treatment: 10 sessions in 3 weeks G1: 10-15 (1rep/5 sec) G2: 100 Hz/50 *μ*s/30 min G3: 6-8 sec/rep, 5 reps (flexion, extension, inclination, rotation) Heat: 20-25 min cervical posterior Follow-up: weeks 3 & 6 post-Tx	VAS ROM elbow extension in median NDT NPQ NPS SF-MPQ	G1 & G2: statistically significant improvement in all the outcomes NPQ, SF-MPQ: G1 > G2 in 3 weeks. (reduced radicular pain & functional improvement) Adverse effects: not reported

Savva (2020)	66 (32/34) [20-75]	Unilateral pain in upper limbs, together with sensory and/or motor symptoms 3 of 4 items +: Spurling, facet distraction, ULTT1, ipsilateral rotation < 60° EMG/ENG: no	G1: cervical traction + neural mobilisation G2: cervical traction + placebo neural mobilisation G3: control	Treatment: 3 ss/week/4 weeks G1: 12-15 min; 10 × 60 sec, rest 30 G2: 12-15 min; 10 × 60 sec, rest 30 G3: no Follow-up: ending	NDI NPRS PSFS Pressure strength Cervical ROM	Statistically significant improvement of the groups in all the outcomes except for pressure strength, active flexion, & inclinations G1 > G3: NDI, NPRS, & contralateral rotation G1 > G2: NPRS

Shafique (2019)	31 (12/19) [20-60]	3 de 4 items +: Spurling, facet distraction, ULTT1, ipsilateral rotation < 60° EMG/ENG: no	G1: spinal mobilisation with movement of arm + conventional Tx G2: conventional Tx: neurodynamics + manual traction	Treatment: 2 ss/3 weeks G1: cervical mobilisation-transversal gliding (10 reps in 1^st^ ssn) (30 reps/3 series in following ssn) G2: conventional Tx: heat (10 min) + active movement in ROM (10 reps/3 series) + isometrics (6-10 sec/20 reps) Neurodynamics: 10 gliding exercises/ssn Manual traction: 10 sec/5 sec rest (10 min) Follow-up: finalisation	NPRS NDI ROM	G1 > improvement in the 3 variables. Statistically significant

Waqas (2016)	100 (63/37) [20-60]	Referred by surgeon or traumatologist for unilateral cervical radiculopathy, due to a disk prolapse. Spurling test, ULTT, cervical distraction, & positive evocation tests EMG/ENG: no	G1: thoracic manipulation G2: cervical mobilisation Both: cervical traction & exercises	Treatment: 3 ss/4 weeks G1: no specific dose G2: no specific dose Cervical traction: 10 min Follow-up: weeks 2 & 4	NDI NPRS	Both groups experience statistically significant improvement in the 2 outcomes. G1 obtained better, statistically significant results

Young (2019)	43 [18-65]	3 de 4 items +: Spurling, facet distraction, ULTT1, ipsilateral rotation < 60° EMG/ENG: no	G1: thoracic manipulation G2: placebo manipulation	Treatment: 1 ssn Follow-up: finalisation & 48/72 h post-Tx	NPRS NDI ROM FME GROC	G1 > G2 neck NPRS not in arm G1 > G2 in NDI, FME, GROC & ROM except inclination to asymptomatic side

## Data Availability

The data used to support the findings of this study are available from public databases, and more details also can be obtained from the corresponding author on request.
